# Anti-thyroid peroxidase antibody and vitiligo: a controlled study

**DOI:** 10.1186/1471-5945-6-3

**Published:** 2006-03-10

**Authors:** Maryam Daneshpazhooh, Mahtab Mostofizadeh G, Javad Behjati, Maryam Akhyani, Reza Mahmoud Robati

**Affiliations:** 1 Department of Dermatology, Tehran University of Medical Sciences, Tehran, Iran; 2 Department of Endocrinology, Tehran University of Medical Sciences, Tehran, Iran

## Abstract

**Background:**

Vitiligo is an acquired depigmenting disorder due to destruction of melanocytes. Although many theories have been suggested for its pathogenesis, the role of autoimmunity is the most popular one. The association of vitiligo with autoimmune thyroid diseases and the increased prevalence of autoantibodies including thyroid autoantibodies in vitiligo favor this role. Our objective was to compare the frequency of thyroid peroxidase antibody (anti-TPO) in vitiligo patients with healthy subjects in Iran.

**Methods:**

Ninety-four cases of vitiligo (46 female and 48 male) and 96 control subjects (49 female and 47 male) were enrolled in this controlled study. Patients with known thyroid disease, history of thyroid surgery and those receiving thyroid medications were not included. The two groups were matched regarding gender and age. The demographic data, symptoms related to thyroid diseases and results of skin and thyroid examinations were recorded in a questionnaire for each subject. Thyroid function tests including free T3, free T4 and TSH-IRMA were performed. Anti-TPO levels were assessed as well. The collected data were analyzed by SPSS version-11 in vitiligo patients and subgroups according to gender, age, extent, and duration of the disease compared with the control group.

**Results:**

Anti-TPO was detected in 17 (18.1%) of patients affected by vitiligo, while this figure was 7 (7.3%) in the control group; the difference was significant with p-value < 0.025 (Phi & Cramer's V = 0.162). When analyzing subgroups, the difference in the frequency of anti-TPO remained significant only in females (p-value < 0.044) (Phi & Cramer's V = 0.207) and in patients in the age ranges of 18–25 (p-value < 0.05) (Phi & Cramer's V = 0.28) and 26–35 year-old (p-value < 0.042) (Phi & Cramer's V = 0.304).

The difference of the frequency of anti-TPO was not significant regarding the duration and extent of vitiligo. In addition, there was no significant difference in the levels of free T3, free T4, and TSH in vitiligo patients compared with the control group.

**Conclusion:**

According to our study, anti-TPO was shown to be significantly more common in vitiligo patients especially in young women, compared with control group. As this antibody is a relatively sensitive and specific marker of autoimmune thyroid disorders including Hashimoto thyroiditis and Graves' disease, and considering the fact that vitiligo usually precedes the onset of thyroid dysfunction, periodic follow-up of vitiligo patients for detecting thyroid diseases is further emphasized especially in young women with increased level of anti-TPO.

## Background

Vitiligo is an acquired depigmenting disorder due to destruction of melanocytes and the resultant absence of pigment production affecting skin and mucosal surfaces with a prevalence of about 1%. Different theories regarding its pathogenesis have been put forward, autoimmunity being the most popular one. The latter is based mainly on the association of vitiligo with known autoimmune diseases and the presence of organ specific antibodies in affected patients [1]. Thyroid functional disorders and autoimmune thyroid diseases have been reported in association with vitiligo and it seems that the incidence of clinical and subclinical thyroid involvement is more common in vitiligo patients than healthy subjects [2]. Vitiligo frequently precedes the thyroid involvement; thus screening vitiligo patients for thyroid function and thyroid antibody seems plausible [3]. Moreover, increased risk of autoimmune/endocrine diseases was shown in first and second degree relatives of vitiligo patients with positive organ specific antibodies [4]. Hashimoto thyroiditis and Graves' disease are the most important and prevalent autoimmune thyroid diseases associated with vitiligo. Elevated levels of anti-TPO are seen in more than 90% cases of Hashimoto thyroiditis and about 75% of Graves' disease cases. This figure is only 10% in healthy people although it may reach 30% in the elderly [5,6]. In this study, we assessed the frequency of anti-TPO as a sensitive marker of autoimmune diseases of the thyroid in vitiligo patients and compared it with healthy subjects in order to find data further supporting the autoimmune theory in the pathogenesis of vitiligo.

## Methods

We conducted this controlled study for comparison of anti-TPO in vitiligo and healthy subjects in Razi Hospital, a university hospital in Tehran, Iran, from September 2004 till March 2005. Ninety-four vitiligo (46 females, 48 males) patients without a history of thyroid surgery or taking medication for thyroid diseases were enrolled. The control group (96 cases; 49 females, 47 males) comprised healthy medical students, medical staff and outpatients' relatives; cases with any history or sign of vitiligo and positive family history of vitiligo in their first and second degree relatives as well as those with a history of thyroid surgery or taking medication for thyroid diseases were excluded. All the subjects in the two groups underwent a complete skin and thyroid examination. The thyroid function tests (Free T4, Free T3, and TSH) and anti-TPO were assessed for all. Radio-immunoassay was used for thyroid function tests (Kits for Free T3 and Free T4: Monobind; TSH: Immunotech). Anti-TPO was assessed using enzyme linked immunosorbant assay (ELISA) (Kit from Monobind) (Normal range: up to 40 IU/ml). The study was approved by the Committee of Ethics of the vice -chancellor of Tehran University of Medical Sciences. Consent was obtained from all participants. The data were analyzed using SPSS software version 11 with Mann-Whitney U, t, chi-square, Fisher's exact, Kolmogorov-Smirnov and Phi & Cramer's V tests.

## Results

Some of the demographic and clinical findings of vitiligo patients are presented in table 1. The mean ages were 28.67 year old (SD = 15.42) and 27.64 year old (SD = 13.7) in the case and control groups, respectively. Thyroid size was normal in 80% (76 cases) of study group versus 83% (73 cases) in control group. The thyroid size was in stage I in 17.9% (17 cases) and 15.9% (14 cases) in the study and case groups, respectively. Two cases (2.1%) of the case group and one case (1.1%) of the control group had stage II thyroid size. The difference of thyroid size was not significant between the two groups (chi-square p = 0.81). The FT3 and FT4 were reported in normal range in 93.6% and 94.7% of study group, and 99% and 96.9% of control group, respectively (FT3 normal range: 1.5–5 pg/ml; FT4 normal range: 0.6–2.6 ng/ml). No significant difference was detected in this regard between the two groups (chi-square p > 0.01). TSH level was significantly higher in the study group than the control one, 1.59 mIU/ml (SD = 1.25) versus 1.14 mIU/ml (SD = 1.48), respectively (Mann-Whitney, p = 0.001) but there was no significant difference between the frequency of normal TSH between the two groups [92.6% versus 86.5% (chi-square p = 0.355)] (TSH normal range: 0.2–5 mIU/ml).

Anti-TPO was detected in seventeen cases (18.1%) in vitiligo patients compared with 7 cases (7.3%) in the control group: The difference was statistically significant with a p-value of 0.025. The intensity of the association of the presence of anti-TPO with vitiligo was 16.2% (Phi & Cramer's V = 0.162). Five cases (10.4%) of males in the vitiligo group were anti-TPO positive while this figure was 2 (4.3%) in the control group with no significant difference (Fisher's exact test: p = 0.226). Twelve female cases (26.1%) in the study group had anti-TPO compared to 5 cases (10.2%) in the control group. There was a significant difference in anti-TPO positivity in female cases between the study and control groups (chi-square = 0.044)  ( Phi & Cramer's V = 0.207). In order to assess the variation of anti-TPO with age, each group was divided into four equal subgroups: lower or equal to 17 year old, between 18 and 25, between 26 and 35 and equal or greater than 36 year-old. In the age range of 18 to 25, 20.8% of the study group and 3.2% of the control subjects had positive anti-TPO results showing a significant difference (Fisher's exact test: p< 0.05) ( Phi & Cramer's V = 0.28). Likewise, 18.5% of vitiligo patients in the age range of 26 to 35 versus 0% of controls showed positive anti-TPO with a significant difference (Fisher's exact test = 0.042) ( Phi & Cramer's V = 0.304). No significant difference was noted in the two other age ranges. Furthermore the frequency of positive anti-TPO showed no significant difference regarding the extent of the disease, the age of onset, the mean duration of disease and its clinical manifestations (including generalized versus non-generalized). Demographic and clinical findings of vitiligo patients with and without anti-TPO are presented in table 2. As only one patient suffered from segmental vitiligo, comparison of segmental and non-segmental vitiligo was not valuable. None of the patients were known cases of other autoimmune diseases.

Clinical autoimmune thyroid disease was diagnosed subsequent to positive anti-TPO results in 4 out of 24 (17%), 2 in the case and 2 in the control group. There were one case of autoimmune thyroiditis (45-year-old woman, anti-TPO positive), one case of Graves' disease (19-year-old woman, anti-TPO positive) and a case of subclinical hypothyroidism (29-year-old man, anti-TPO negative) in the vitiligo group and two patients with Hashimoto thyroiditis (41-year-old woman and 12-year-old boy, both anti-TPO positive), one case of transient thyroiditis (30-year-old man, anti-TPO negative) and one patient with subclinical hypothyroidism (33-year-old man, anti-TPO negative) in the control group.

## Discussion

Vitiligo is a generalized autoimmune disease manifesting acquired white patches due to loss of melanocytes. It is the most prevalent pigmentary disorder with an incidence rate between 0.1–2% showing multifactorial etiology and polygenic inheritance [7]. The exact etiology and pathogenesis of this disease is not clear. Many theories have been put forward in this regard, autoimmune theory is the most popular one. Increased prevalence of autoimmune disorders in association with vitiligo, detection of various autoantibodies including anti-thyroid and anti-melanocyte antibodies in the serum of vitiligo patients and alteration of T-cell population showing decreased T-helper cells are in favor of this role [1].

Various thyroid autoantibodies including thyroid stimulating antibody, anti- thyroglobulin antibody and anti-thyroid peroxidase antibody, are detectable in autoimmune thyroid diseases the latter being the most sensitive test for the diagnosis and follow-up of this group of diseases. Thyroid peroxidase is responsible for the iodination of tyrosine residues in the thyroglobulin molecule. Anti-TPO antibody has been shown to mediate thyroid cell destruction in vitro. This antibody, historically referred to as the anti-microsomal antibody, is established as a sensitive tool for the detection of early subclinical autoimmune thyroid diseases, follow up of the response to immunotherapy and identification of at-risk cases for autoimmune thyroid diseases [8].

Clinical as well as functional abnormalities of the thyroid gland have been reported by many authors to be significantly more frequent in vitiligo patients compared with control group [2,4]. We didn't find any significant difference in thyroid size and thyroid function test results between the case and control groups, although the plasma level of TSH was significantly higher in vitiligo patients but this increase remained in the normal range. This difference may be due to the lower mean age of patients in our study since older cases are shown to be more at risk of evolution toward subclinical thyroid diseases,[9] although Iacovelli and his colleagues found a significant frequency of thyroid diseases in children with non-segmental vitiligo [10].

According to our study the rate of positive anti-TPO was significantly higher in vitiligo patients compared with control subjects. Mandry et al assessed the presence and frequency of organ specific antibody in 20 patients with vitiligo and their relatives. They detected anti-microsomal and anti-thyroglobulin antibodies in 50% and 40% of their cases, respectively; they showed increased prevalence of organ specific antibodies in the relatives, as well [9]. Morgan et al also found higher prevalence of thyroid antibodies in vitiligo patients, especially in generalized vitiligo, compared with healthy people [11]. Dave et al showed antibody positivity (anti-thyroglobulin, anti-TPO) in 31.4% of their cases in India against 10% of their controls [12]. Grimes, Korkij, and Betterle also showed high frequency of antithyroid antibodies [4,13,14]. Kurtev and Dourmishev reported anti-microsomal antibodies in 50% of their cases with vitiligo (children and adolescents) [15]. Iacovelli et al reported a figure of 7% in children with non-segmental vitiligo, especially in females, all of them had thyroid dysfunction [10].

According to our study, the difference in the prevalence of anti-TPO was significant only in female cases and patients in the age ranges of 18 to 35 year-old, findings not previously reported in the literature. In our study we found no relationship between the presence of anti-TPO antibodies and the extent, duration, age of onset and anatomical location. Morgan found autoantibodies especially in generalized vitiligo [11], Mandry in patients with later onset of the disease and a higher mean age [9], Betterle in patients with long lasting vitiligo,[14] and Dave in patients with mucosal and early onset vitiligo [12]. Further studies with larger sample size are suggested to elucidate these issues in the future.

## Conclusion

According to our study, anti-TPO was shown to be significantly more common in vitiligo patients especially in young women, compared with control group. As this antibody is a sensitive tool for the detection of autoimmune thyroid disorders including Graves' disease and Hashimoto thyroiditis, and considering the fact that vitiligo usually precedes the onset of thyroid dysfunction, periodic follow-up of vitiligo patients for detecting thyroid diseases is further emphasized especially in young women with increased level of anti-TPO.

## Competing interests

The author(s) declare that they have no competing interests.

## Authors' contributions

DM conceived the study. DM, MM, AM and BJ participated in its design and coordination. RRM and MM performed the data collection. MM and DM participated in the statistical analysis. DM and RRM drafted the manuscript. All authors read and approved the final version of the manuscript.

## Pre-publication history

The pre-publication history for this paper can be accessed here:



## Figures and Tables

**Table 1:** Clinical characteristics of vitiligo patients

Mean age +/- SD (year)	28.67+/-15.42
Age range	4–83
Gender (female:male)	63:31 (2:1)
Age (year)	
=>17	22 (23.4)
18–25	24 (25.5)
26–35	27 (28.7)
=<36	21 (22.3)
Mean age of onset +/- SD (year)	22.86+/-15.37
Age of onset range(year)	2–82
Mean duration +/- SD (year)	5.85+/-7.55
Duration Range (year)	0–40
Extent (%)	
<20	70 (74.5)
20–50	20 (21.3)
>50	3 (3.2)
Type of vitiligo, n (%)	
Generalized	63 (67)
Focal	17 (18.1)
Acrofacial	5 (5.3)
Mucosal	2 (2.1)
Segmental	1 (1.1)
Lip-tip	1 (1.1)
Universalis	1 (1.1)
Unclassified	4 (4.3)

**Table 2:** Comparison of clinical features of vitiligo patients with and without anti-thyroid peroxidase antibody

Clinical features	No. (%) with autoantibodies (17 patients)	No. (%) without autoantibodies (77 patients)
Mean age +/- SD, yr	26.47 +/-11.52	29.16 +/- 16.18
Gender		
Male	5 (10.4)	43 (89.6)
Female*	12 (26.1)	34 (73.9)
Age (year)		
=>17	4 (18.2)	18 (81.8)
18–25*	5 (20.8)	19 (79.2)
26–35*	5 (18.5)	22 (81.5)
=<36	3 (14.3)	18 (85.7)
Mean onset age +/- SD,yr	19.59 +/- 9.5	23.59 +/-16.36
Mean duration +/- SD,yr	6.88 +/- 9.9	5.62 +/- 6.98
Extent (%)		
<20	12 (17)	58 (83)
20–50	5 (25)	15 (75)
>50	0	3 (100)
Type of vitiligo		
Generalized	13 (20.6)	50 (79.4)
Non-generalized	4 (12.9)	27 (87.1)

*Difference significant compared with the control group (pv < 0.05)
